# Lonely in Lockdown: Predictors of Emotional and Mental Health Difficulties Among Jewish Young Adults during the COVID-19 Pandemic

**DOI:** 10.1007/s12397-021-09373-3

**Published:** 2021-06-08

**Authors:** Graham Wright, Sasha Volodarsky, Shahar Hecht, Leonard Saxe

**Affiliations:** 1 Brandeis University, 415 South Street, MS 014, Waltham, MA 02453-2728 USA; 2 Northeastern University, Boston, USA

**Keywords:** Jewish young adults, Mental health, COVID-19, Pandemic, Birthright Israel, Emerging adults, Loneliness, College students

## Abstract

As individuals undergoing a developmental process characterized by identity exploration, Jewish young adults are particularly vulnerable to the disruption of social connections related to the COVID-19 pandemic. Recent research has demonstrated that young adults, including young Jews, have experienced higher rates of mental health difficulties than older individuals during the pandemic. Using data from a survey of Jewish young adults who applied to participate in Birthright Israel summer 2020 trips but were unable to participate due to the pandemic, we examined the factors contributing to young adults’ mental health difficulties. We found that loneliness, rather than financial worries or concerns about the health impacts of COVID-19, was the single most important driver of reported emotional or mental health difficulties. Results also suggested that simply increasing the frequency of contacts between individuals is unlikely to reduce loneliness, unless these are positive, substantial connections, such as those among members of a “social support network.” Building and rebuilding deep, meaningful social connections between Jewish young adults should be a top priority for Jewish organizations going forward.

## Introduction

As the enormity of the COVID-19 pandemic continues to unfold, the magnitude of the health crisis and its attendant economic and social effects have come into sharp relief. Prior to the release of vaccines in early 2021, social distancing was one of the most effective means of controlling the spread of the virus. Consequently, governments closed or limited the use of venues where individuals gathered, including schools, workplaces, restaurants, theaters, and religious institutions. For Jews, the restrictions on congregating posed special challenges, given religious, cultural, and communal traditions that prioritize face-to-face interactions. The psychological impact of long-term isolation and social distancing threatened to be especially acute for young adults, who were in the process of constructing their adult identities and building lasting social connections (Arnett et al. 2014).

This study is designed to understand the different ways that the pandemic has impacted the psychological well-being of Jewish young adults. We explore whether economic stressors, COVID-related anxieties, the disruption of social networks and interpersonal connections, or lost opportunities of involvement in Jewish life influenced mental health difficulties experienced by Jewish young adults. We also explore whether these relationships were mediated through increased loneliness. These results provide important context for understanding Jewish young adults’ experiences of the pandemic, and the potential role of Jewish programs and institutions in responding to their needs.

## The Social Disruptions of COVID-19

During 2020, COVID-19 was responsible for nearly 350,000 deaths in the United States, with many occurring in densely populated cities like New York (Woolf et al. 2020; Ahmad and Anderson 2021). American Jews, concentrated in Northeast urban areas (Saxe et al. 2021), were in a particularly vulnerable position to be affected in the pandemic’s early spread. Aside from the direct effects of the virus and the economic impact of efforts to curb its spread, widespread social distancing enacted to control the spread of the virus had troubling implications for the American Jewish population, given the importance of the Jewish community’s social and communal dimensions (Aronson et al. 2019; Hartman and Sheskin 2012).

The potential for the pandemic to sever social connections had especially important implications for young adult Jews, members of the millennial and Gen Z cohorts. Psychologically, this period of “emerging adulthood” is characterized by identity exploration (Arnett 2014), which can lead to anxiety and depression even in “normal” times (Arnett et al. 2014). Prior to the pandemic, in recognition of the unique issues of this period of life, the Jewish community had developed a host of initiatives for young adults, most of which focused on building personal connections. Prominent among these programs was Birthright Israel, which has enabled nearly 500,000 Jewish young adults from the United States to travel with peers to Israel over the past two decades (Saxe and Chazan 2008; Wright et al. 2020). Other Israel engagement programs, including MASA,^1^ Moishe House,^2^ and OneTable,^3^ as well as Hillel, Chabad, and other campus-based initiatives (Koren et al. 2016), also promoted social interactions between Jewish young adults in myriad ways. Because person-to-person contact was an essential element in all of these programs, the pandemic forced most to suspend operations or to function online.

Social disruptions associated with the pandemic extended beyond denying Jewish young adults opportunities to connect with the Jewish community and one another. A study of Jews in 10 US communities during the summer of 2020 documented many of the health and emotional effects of the pandemic (Aronson et al. in press). The study confirmed that Jewish young adults were particularly affected by the significant disruptions to social interactions in each of the communities. These disruptions of social connections not only prevented young Jews from maintaining connections with Jewish institutions and members of the Jewish community, but also contributed to serious mental health challenges. Jews between the ages of 18 and 34 were more likely to report experiencing emotional or mental health difficulties, to report being lonely, and to experience difficulty coping with the pandemic, compared to older Jews, and these outcomes were not a result of differences in financial status.

## Understanding the Causes of Mental Health Challenges Among Young Adults During COVID-19

Recent research has confirmed that young adults disproportionately experienced mental health challenges due to the pandemic. A Pew Research Center study of the general American population found that during the pandemic, Americans aged 18–29 had the highest reported rates of emotional distress (Keeter 2020). Other research found that although younger Americans expressed less anxiety about becoming sick with COVID-19, they reported more warning signs for depression and anxiety during the pandemic than their older peers (Bruin 2021). These results are especially troubling because there was already great cause for concern about the mental health situation of young adults. Prior to the pandemic, commentators and mental health professionals spoke of a mental health “crisis” on American college campuses (Hibbs and Rostain 2019), supported by data finding a growing demand for mental health services among US undergraduates (Lipson et al. 2019). Other recent studies of student life at universities with large populations of Jewish students also identified loneliness, stress, and mental health among the top challenges facing students (Shain et al. 2016; Wright et al. 2019a, b).

Effectively responding to these crises, however, requires a deeper understanding of the specific factors driving those challenges. Existing research suggests that there are a number of distinct mechanisms by which the pandemic *could* have exacerbated mental health problems for young Jews, each of which recommends distinct responses. Perhaps the most obvious driver of mental health challenges during the pandemic was widespread loneliness, which in turn was driven by the disruption of social relationships necessitated by social distancing. Liu et al. (2020) found that loneliness was a key predictor of depression, anxiety, and post-traumatic stress disorder among American young adults during the pandemic, but also that increased social connections with parents or significant others helped alleviate mental health difficulties. Similarly, Lee et al. (2020) found an increase in depression among young adults during the pandemic and argued that much of this increase could be explained by a coincident increase in loneliness.

Aside from its impact on social interactions, the pandemic may have impacted the mental health of Jewish young adults in other ways. One obvious driver of mental health challenges might be increased anxieties related to the health impacts of the virus itself. Liu et al. (2020) found that concerns about becoming ill or spreading the virus to others were associated with depression and anxiety, even after controlling for loneliness and social support. Research also found that the pandemic’s economic disruptions, including job losses, could also negatively impact mental health well-being (Posel et al. 2021).

Paradoxically, the numerous opportunities for social connections that existed for Jewish young adults *before* the pandemic, which were subsequently interrupted or disrupted, may have led to an even greater sense of isolation. For young adults in general, the steepest decline in mental health during the pandemic appears to have been among those young adults who were *not* accustomed to dealing with isolation and loneliness, rather than among those who were unengaged in social activities (Hamza et al. 2020). Thus, those young Jews who had previously been involved in programs that foster social relationships, such as those sponsored by Hillel, Moishe House, or OneTable, may have been especially affected by their absence. The suspension of Birthright Israel trips in April 2020, after approximately 23,000 American young Jews had already applied to participate in summer 2020 trips, represented another loss of potential social connections. These Birthright applicants, who expected to have an intensive 10-day experience with American and Israeli Jewish peers, instead found themselves, like many other Americans, deprived of an important opportunity for meaningful social engagement.

The specific goal of this paper is to understand the different ways in which the pandemic has impacted the mental health of Jewish young adults. In light of previous research, we hypothesize that financial concerns and job loss, concerns about the health impacts of COVID-19, and involvement with Jewish activities before the pandemic will all be positively associated with experiencing mental health difficulties during the pandemic. We also hypothesize that a greater frequency of virtual and in-person social interactions during the pandemic and having a robust social support network will be negatively associated with experiencing mental health difficulties. In addition, we hypothesize that loneliness will not only be a significant positive predictor of mental health difficulties, but that it will also mediate the relationship between experiencing mental health difficulties and other factors. That is, we hypothesize that these factors are positively or negatively associated with experiencing mental health difficulties partly due to their impact on loneliness itself. These hypotheses are tested using a dataset of Jewish young adults who applied to participate in Birthright Israel summer 2020 trips but were unable to participate due to the suspension of trips.

## Data and Methods

Data for this study come from a pair of online surveys of US Jewish young adults between the ages of 18 and 32 who applied to Birthright Israel’s summer 2020 trips. Because these trips were canceled prior to the survey, none of these applicants participated in a Birthright trip. The survey was emailed to two independent random samples, each consisting of 7200 applicants (out of a total population of 23,000). The first survey was fielded during September 2020 and achieved a response rate of 15.8%.^4^ The second survey was fielded in February 2021 and achieved a response rate of 14.6%.

Our dependent variable was the frequency at which respondents reported experiencing emotional or mental health difficulties during the last week. This item was adapted from the long-running “Healthy Minds” survey assessing mental health challenges among college students (Eisenberg and Lipson 2019). The original question had five categories (“never,” “rarely,” “sometimes,” “often,” and “all the time”). Because relatively few respondents gave the answer “all the time,” we collapsed the top two categories so the resulting variable had four categories (“never,” “rarely,” “sometimes,” “often/all the time”). Our independent variables were loneliness, economic stressors during the pandemic, anxiety related to the health impacts of COVID-19, level of interpersonal social connections, perceived size of social support network, and pre-pandemic involvement in Jewish life. We measured loneliness with a question adapted from surveys conducted by the Pew Research Center (Keeter 2020) and asked respondents how often they felt lonely in the past week, using the same answer categories as the mental health difficulties question. Once again, relatively few respondents reported being lonely “all the time,” and so the top two categories were collapsed to create a four-category ordinal scale (“never,” “rarely,” “sometimes,” “often/all the time”).

Economic stressors during the pandemic were measured using an index that accounted for subjective financial worries^5^ as well as an indicator of current employment status. To measure anxieties related to the health impacts of COVID-19, questions were included that assessed concern over becoming seriously ill and spreading the virus to others. The role of interpersonal social connections was measured through a variety of subjective and objective measures, including living situation, and two separate indices that summarized frequency of virtual and in-person social interactions.^6^ To measure social support networks, we adapted a question from a Kaiser Family Foundation survey on loneliness and social isolation (DiJulio et al. 2018), asking respondents how many friends or relatives they had living nearby whom they could rely on for help and support. To assess involvement in communal Jewish life before the pandemic, we included a variable measuring self-reported pre-pandemic frequency of participation in activities sponsored by Jewish organizations.

All models also included controls for being an undergraduate student and survey date (September 2020 or February 2021). As existing research indicated that young adults who identify as female or LGBTQ were more likely to report mental health struggles, the models included controls for gender and identifying as LGBTQ (Wright et al. 2019a, b; Perlis et al. 2020). Other research focusing on the link between mental health and religion argued that Orthodox Jews were more insulated from the mental health effects of the pandemic due to their stronger religiosity (Pirutinsky et al. 2020), and that atheists and agnostics had lower levels of psychological well-being compared to those with formal religious affiliations (Hayward et al. 2016). We therefore included a measure of Jewish denomination that distinguished between four groups: those who identify as Orthodox, those who identify as secular/culturally Jewish, those who identify as “Just Jewish” with no particular denomination, and those who identify with another Jewish denomination including Reform, Conservative, Reconstructionist, Renewal, and Humanist.^7^ Descriptive statistics for all variables are shown in Table 1.

**Table 1:** Descriptive statistics (unweighted)

		N	%/Mean (SD)
Frequency of emotional or mental health difficulties (last week)	Never	586	19
	Rarely	827	27
	Sometimes	908	29
	Often/All the time	759	25
Frequency of feeling lonely (last week)	Never	362	12
	Rarely	692	22
	Sometimes	1194	39
	Often/All the time	839	27
Current employment status	Working	1878	58
	Not working, looking for work	651	20
	Not working, not looking	682	21
How concerned are you that you might spread the coronavirus to other people?	Not at all concerned	207	6
	Not too concerned	635	20
	Somewhat concerned	1305	40
	Very concerned	1084	34
How concerned are you that you will get the coronavirus and require hospitalization?	Not at all concerned	693	21
	Not too concerned	1193	37
	Somewhat concerned	891	28
	Very concerned	453	14
Number of people in support network	No one/a few people	1169	38
	A fair number of people	1339	43
	A lot of people	591	19
Current living arrangements	Live with parents	1239	42
	Live with sig. other/Spouse	370	12
	Live alone	297	10
	Live in dorm/ with roommates/Other	1071	36
Jewish religious affiliation	Secular/Culturally Jewish	547	18
	Just Jewish (no particular denomination)	767	26
	Specific denomination	1399	47
	Orthodox/Modern Orthodox	280	9
Frequency of participation in activities sponsored by Jewish organizations (before coronavirus crisis)	Never	758	25
	Once a year/every few months	1606	52
	Once a month/every week	704	23
Gender	Female	1973	61
Do you identify as LGBTQ?	Yes	446	15
Being an undergraduate student	Yes	1707	53
September 2020		1134	35
February 2021		2102	65
Index of financial worries (mean)		3177	8.04 (3.35)
Index for frequency of virtual social interactions (mean)		3081	5.82 (1.36)
Index for frequency of in-person social interactions (mean)		3082	4.5 (1.67)

Our investigation concerned the extent to which economic stressors, COVID-related health concerns, social interactions and support networks, and pre-pandemic levels of engagement with communal Jewish life were associated with recent experiences of mental health difficulties. Because earlier research suggested that loneliness was perhaps the most important driver of mental health challenges, we were also interested in exploring this complex relationship (Lee et al. 2020; Liu et al. 2020). Our examination of this relationship was not limited to the direct relationship between these two variables (loneliness and mental health) but also included an examination of the extent to which factors such as health concerns, social interactions, and Jewish engagement were partly or fully mediated through their impact on loneliness. For example, in-person social interactions may reduce mental health difficulties purely because they lead to reduced loneliness. Similarly, those who prior to the pandemic participated in Jewish activities more frequently may experience more mental health difficulties because the loss of those experiences led to increased loneliness. In these situations, we would not expect variables for in-person social interactions or participation in Jewish activities to be statistically significant in a model of mental health difficulties that also controls for loneliness, unless the variable in question also had an *additional* impact on mental health difficulties, beyond its impact on loneliness.

To address this complexity and to provide a fuller picture of how these factors relate to experiencing mental health difficulties among Jewish young adults, we ran a series of ordered logistic regression models. First, we ran a model of mental health difficulties controlling for the factors discussed above, but *without* controlling for loneliness. This model showed the relationship between these factors and experiencing mental health difficulties, regardless of whether or not these relationships were mediated through loneliness. We then ran a second model of mental health difficulties that added a control for loneliness. This model assessed the impact of loneliness itself, as well as the extent to which other variables had an unmediated impact on experiencing mental health difficulties, after accounting for their impact on loneliness. To allow for a meaningful comparison of coefficients between these two ordered logistic regression models, we used a method developed by Karlson et al. (2012) which adjusts the coefficients for the “restricted” model without the control for loneliness, so that they are calculated on the same scale as the “full” model which includes the loneliness variable. Finally, we ran a third model to identify the factors that were associated with increased loneliness, regardless of their impact on experiences of mental health difficulties. All analyses used weights that corrected for nonresponse bias.^8^

## Results

To illustrate the bivariate relationship between loneliness and experiencing mental health difficulties, Table 2 shows the proportion of respondents with a given level of loneliness who reported different levels of mental health difficulties. As can be seen, there was an extremely strong relationship between the two variables—75% of those who reported “never” feeling lonely in the past week also reported “never” experiencing mental health difficulties, while 61% of those who reported feeling lonely “often/all the time” also reported experiencing mental health difficulties “often/all the time” in the past week.

**Table 2:** Weighted cross-tab of experiencing mental health difficulties in the past week by loneliness in the past week

Frequency experiencing mental health difficulties in the past week	Frequency feeling lonely in past week			
	Never (%)	Rarely (%)	Sometimes (%)	Often/all the time (%)
Never	75	30	9	2
Rarely	19	47	32	9
Sometimes	5	17	45	28
Often/all the time	1	5	14	61
Total	100	100	100	100

Design-adjusted chi-square test significant at *p* < .001

To more fully investigate the relationship between mental health, loneliness, and other key independent variables, we first present a model for experiencing mental health difficulties without a control for loneliness (Table 3, Model 1). This model shows that those with greater financial worries were more likely to experience mental health difficulties, as were those who at the time of the survey were unemployed and looking for work, compared to those who were employed. Young adult Jews with stronger social support networks—who had more people they could rely on for help—were significantly less likely to experience mental health difficulties. Those Jews who identified as Orthodox were significantly less likely to experience mental health difficulties, compared to those who identified as Reform, Conservative, or other non-Orthodox denominations. Women and those who identified as LGBTQ were more likely to experience mental health difficulties. In contrast, concerns about becoming seriously ill from COVID-19 were not significantly associated with mental health difficulties, but concerns about spreading the virus to others were. Having more in-person social interactions was not significantly associated with experiencing mental health difficulties, nor was a person’s living situation or levels of pre-pandemic participation in programs sponsored by Jewish organizations. However, higher frequency of online virtual social interactions was associated with a greater likelihood of experiencing mental health difficulties. Undergraduate students were also significantly more likely to experience mental health difficulties compared to non-undergraduates.^9^ The model also shows higher levels of mental health difficulties in February 2021 compared to September 2020, after controlling for other factors.

**Table 3:** Ordered logit models of experiencing mental or emotional difficulties in the past week

	Model 1		Model 2	
	Coef	Robust SE	Coef	Robust SE
Loneliness	–	–	1.646**	0.064
Financial concerns	0.183**	0.014	0.102**	0.014
*Employment status*				
Employed	–	–	–	–
Unemployed, looking for work	0.496**	0.112	0.231*	0.111
Unemployed, not looking for work	0.050	0.113	−0.014	0.113
Concerns about spreading COVID to others	0.287**	0.057	0.153**	0.056
Concerns about becoming seriously ill with COVID	−0.006	0.053	−0.046	0.053
*Social support network*				
No one/a few people	–	–	–	–
A fair number of people	−0.652**	0.099	−0.103	0.098
A lot of people	−1.215**	0.131	−0.33*	0.130
*Living situation*				
Live alone	–	–	–	–
Parents	0.180	0.149	0.151	0.149
Significant other/spouse	−0.295	0.177	0.662**	0.178
Dorm/roommates/sibling/other	0.244	0.154	0.27	0.154
Virtual social interactions	0.113**	0.033	0.024	0.033
In-person social interactions	−0.021	0.027	0.029	0.027
*Pre-COVID participation in activities sponsored by Jewish org*				
Never	–	–	–	–
Once a year/every few months	0.059	0.103	−0.039	0.103
Once a month/every week	−0.043	0.133	−0.249	0.133
*Jewish denomination*				
Specific denomination	–	–	–	–
Secular/cultural	0.146	0.116	0.265*	0.116
Just Jewish	−0.129	0.105	−0.096	0.106
Orthodox/Modern Orthodox	−1.028**	0.189	−0.498*	0.188
Female	0.514**	0.087	0.175*	0.087
Identifies as LGBTQ	0.633**	0.119	0.298*	0.119
Undergraduate student	0.242*	0.095	0.143	0.095
February 2021 (vs. September 2020)	0.485**	0.085	0.237**	0.085
Observations	2770		2770	

**p < 0.01; *p < 0.05. Coefficients in Model 1 adjusted using KHB correction (Karlson et al. 2012) to allow for direct comparison with coefficients in Model 2

Model 2 added a control for being lonely in the past week and found that, as expected, loneliness was a particularly strong predictor of experiencing emotional or mental health difficulties. Financial worries were still significantly related to experiencing mental health difficulties in the same direction as before, even after controlling for loneliness. Being unemployed and looking for work and having a strong social support network were still associated with mental health difficulties, although the coefficients for these variables were now smaller and only significant at the 95% level, implying that the relationships seen in Model 1 were partly due to higher levels of loneliness among those looking for work and those without strong social support networks. Although it was nonsignificant in Model 1, living with a significant other was associated with a *higher* frequency of experiencing mental health difficulties after controlling for loneliness, suggesting that living with a partner who did *not* make one feel less lonely might be a separate, positive predictor of mental health difficulties. After controlling for loneliness, the negative relationship between Orthodoxy and mental health difficulties remained but was diminished in magnitude and significance, while secular/cultural Jews were significantly more likely to experience mental health difficulties, compared to those who were affiliated with other Jewish denominations. Women, those who identified as LGBTQ, and those who responded to the survey in February 2021 remained more likely to experience mental health difficulties, even after controlling for loneliness. After controlling for loneliness, undergraduate students were no longer significantly more likely to experience mental health difficulties than non-undergraduates. In general, most of the significant effects identified in Model 1 were smaller in magnitude in Model 2, suggesting that loneliness mediated the impact of many different factors that contribute to mental health difficulties.

**Fig. 1:**
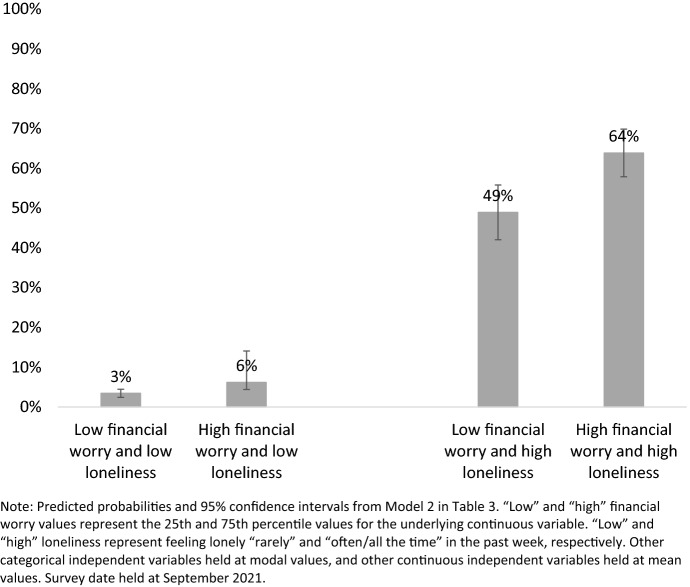
Predicted probability of experiencing mental health difficulties “often/all the time” in the past week by financial worry and loneliness

Because coefficients from ordered logit models have no intuitive interpretation, it is difficult to assess the relative magnitude of the different effects discussed above from the models themselves. To address this, Fig. 1 presents the predicted probability of experiencing emotional or mental health difficulties “often/all the time” in the past week for those with different levels of financial concern and loneliness, as estimated by Model 2 in Table 3. Young Jews who reported being lonely only “rarely” in the past week had a very low predicted likelihood of experiencing mental health difficulties during the same period, regardless of whether they had low or high levels of financial worries. In contrast, young Jews who reported being lonely “often/all the time” had a 49% likelihood of experiencing emotional or mental difficulties "often/all the time" in the past week if they had low levels of financial worries, and a 64% likelihood if they had high levels of financial worries.

Table 4 presents a model of loneliness itself. As implied by the results above, being unemployed and looking for work were associated with greater loneliness, as was having greater financial worries. Having a more robust social support network was associated with decreased loneliness, as was living with a significant other, compared to living alone. Living with family or roommates was not related to loneliness. Concern about becoming seriously ill with COVID-19 was not associated with increased loneliness, but concern about spreading COVID-19 to others was. Having in-person social contacts with others was associated with lower levels of loneliness, while having virtual social contact with others was associated with higher levels of loneliness. Orthodox respondents were less likely to report being lonely compared to those who were affiliated with other Jewish denominations, while those who attended Jewish communal activities at least once a month *before* the pandemic were more likely to report recent loneliness. Women, those who identified as LGBTQ, and those interviewed in February 2021 were more likely to report being lonely. Undergraduates were not significantly more or less likely to report being lonely.

**Table 4:** Ordered logit model of experiencing loneliness in the past week

	Coef.	Robust SE
Financial concerns	0.109**	0.015
*Employment status*		
Employed	–	–
Unemployed, looking for work	0.364**	0.165
Unemployed, not looking for work	0.067	0.111
Concerns about spreading COVID to others	0.165**	0.062
Concerns about becoming seriously ill with COVID	0.059	0.053
*Social support network*		
No one/a few people	–	–
A fair number of people	−0.723**	0.047
A lot of people	−1.134**	0.041
*Living situation*		
Live alone	–	–
Parents	0.051	0.153
Significant other/spouse	−1.204**	0.050
Dorm/roommates/sibling/other	−0.049	0.139
Virtual social interactions	0.114**	0.036
In-person social interactions	−0.056*	0.025
*Pre-COVID participation in activities sponsored by Jewish organizations*		
Never	–	–
Once a year/every few months	0.131	0.116
Once a month/every week	0.264*	0.165
*Denomination*		
Specific denomination	–	–
Secular/cultural	−0.151	0.102
Just Jewish	−0.017	0.104
Orthodox/Modern Orthodox	−0.665**	0.085
Female	0.437**	0.129
Identifies as LGBTQ	0.456**	0.184
Undergraduate student	0.13	0.104
February 2021 (vs. September 2020)	0.332**	0.114
Observations	2776	

**p < 0.01; *p < 0.05

**Fig. 2:**
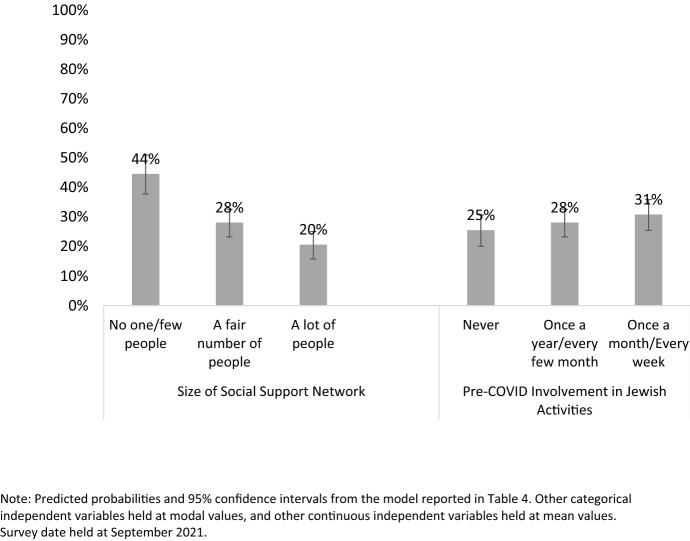
Predicted probability of feeling lonely “often” or “all the time” in the past week, by social support network and prior participation in Jewish-sponsored activities

To illustrate the relative magnitude of the effects for social support network and pre-COVID Jewish involvement, Fig. 2 shows the estimated probability of being lonely “often/all the time” in the past week, derived from the model reported in Table 4. A “typical” respondent who reported that they had “no one/only a few people” living near them who could provide support had an estimated 44% probability of being lonely “often/all the time” in the past week, compared to only 20% for a similar respondent who reported having “a lot of people” nearby who could help. The impact of pre-pandemic levels of participation in programs sponsored by Jewish organizations was smaller in magnitude, but still notable. Holding all else (including social support network) constant, a respondent who reported participating in Jewish-sponsored activities at least monthly before the pandemic had a 31% probability of being frequently lonely during the past week, compared to 25% for a similar respondent who never participated in Jewish activities.

## Discussion

The present study provides a snapshot of the impact of the COVID-19 pandemic on the emotional and mental health difficulties experienced by American Jewish young adults in the summer of 2020 and the winter of 2021. By disrupting social connections during a critical period in young adults’ emotional and psychological development, the pandemic, and the necessary public health requirements that disrupted in-person gatherings, clearly had negative consequences for young adults’ mental health. Among our sample of Jewish adults aged 18–32, loneliness was the single most important driver of experiencing emotional or mental health difficulties through the summer of 2020, and the results suggest that the situation deteriorated even further by the winter of 2021. Concerns about becoming sick with COVID-19 were not significantly related to mental health difficulties and, although financial worries, concerns about spreading the virus, and frequency of social interactions were significant predictors, their impact appeared to be dwarfed by that of loneliness. Indeed, some of these factors may have influenced mental health difficulties primarily through their impact on loneliness. For example, being unemployed and looking for work (as opposed to being currently employed) appeared to lead to mental health difficulties largely because it was associated with increased loneliness, presumably due to the loss of social connections with coworkers. Likewise, Orthodox respondents were also less likely to report mental health difficulties than those who identified with other Jewish denominations, partly because they were less likely to feel lonely. Those with stronger social support networks were less likely to experience mental health difficulties, although results also suggest that robust social networks may have bolstered mental health in other ways, aside from their impact on loneliness. These findings suggest that the top priority for efforts to address mental health difficulties among Jewish young adults should be to provide opportunities for them to build or rebuild social networks.

Perhaps the most interesting finding, with implications beyond those associated with the pandemic, is that, in terms of addressing loneliness, not all social interactions were equal. Having in-person social interactions was significantly associated with lower levels of loneliness, while having more frequent virtual interactions was significantly associated with *higher* levels of loneliness. The most likely explanation for this finding is that those who were already more lonely were more likely to seek out online connections, but that these online interactions did little to actually alleviate loneliness (Amichai-Hamburger and Ben-Artzi 2003). More generally, these results suggest that conversations or social gatherings with friends or family—virtual or otherwise—were unlikely, on their own, to dramatically reduce loneliness or mental health difficulties among young Jews.

Similarly, our results suggest that although those living with a spouse or significant other tended, unsurprisingly, to be less lonely than those living alone, being forced to endure “lockdown” with a partner who did *not* reduce a feeling of loneliness could actually exacerbate mental health difficulties. As implied by the results of Hamza et al. (2020), we also found higher levels of loneliness among those who before the pandemic participated more frequently in Jewish communal activities. That the *loss* of opportunities to connect with other Jews appears to be a driver of loneliness points to the importance of these opportunities for Jewish young adults.

Simply increasing the frequency of contact between individuals, or offering more opportunities to connect, seems unlikely to dramatically reduce the loneliness felt by many Jewish young adults. Rather, positive, substantial connections, such as those with members of a social support network, appear to be more important. Our findings also highlight a number of subgroups of Jewish young adults who appeared at especially high risk of experiencing mental health challenges, for reasons not necessarily related to loneliness. Echoing earlier research on young adults from before the pandemic (Perlis et al. 2020; Wright et al. 2019a, b) we found that women and those who identified as LGBTQ were more likely to report mental health difficulties, even after accounting for loneliness, although both groups were *also* more likely to report being frequently lonely. Jews who identified as “secular/cultural” were also more likely to report mental health difficulties, compared to those affiliated with other denominations, despite the fact that they were *not* more likely to report being lonely. As argued by past work, this could reflect the lack of “psychological coping resources” that may be provided by religious belief (Hayward et al. 2016; Pargament 1997). At the same time, despite continued concerns about mental health challenges among undergraduates, we found no evidence that undergraduate Jews were lonelier or more likely to experience emotional or mental health difficulties compared to other Jewish young adults with similar characteristics.

One potential limitation of the present study is that our sample was drawn from applicants to Birthright Israel. Those who explicitly sought out an intensive group Jewish experience may have been more in need of personal connections than others. Their expectation of a group experience and distress over its loss may also have heightened their need for social interaction. However, given the size and diversity of the Birthright Israel applicant pool (Wright et al. 2019a, b) it seems implausible that the 23,000 American young Jews who applied to Birthright Israel in 2020 had dramatically different mental health needs than their Jewish peers who did not.

## Conclusion

As they enter and navigate “emerging adulthood” (Arnett 2014), young Jews seek to explore and define their own sense of identity through their connections with one another. Forging and maintaining these connections, a difficult task even in the best of times, may be all but impossible during a period of enforced isolation and social distancing. Through its impact on social interactions, including the involvement with Jewish organizations and programs, COVID-19 represents a serious shock to the emotional and mental health of young American Jews. Although the Jewish community was already focused on building connections between young Jews before the pandemic, the current need for community appears to be higher than ever before.

## Footnotes

1 https://www.masaisrael.org.

2 https://www.moishehouse.org.

3 https://onetable.org.

4 Response rates are calculated according to the American Association of Public Opinion Research’s (AAPOR) “RR2” formula, which includes partial responses but does not include a correction for unknown eligibility.

5 The index was created by adding together responses to the items in the following question bank: How worried are you that… “You will not be able to afford to pay for your basic living expenses,” “You will not be able to afford health care,” “You will not be able to afford the standard of living you are accustomed to,” “You will not have enough money for your retirement.” Answer options were (1) “not at all worried”, (2) “not too worried”, (3) “somewhat worried”, (4) “very worried.”

6 Each index was created by summation of the values of two questions. The in-person social interaction questions were “Last week, how often did you have a conversation IN PERSON with a family member or close friend not living with you?” and “In the past month, how often have you had a party or social gathering IN PERSON with friends or family who don't live with you?” The virtual social interactions questions were “Last week, how often did you have a conversation by PHONE OR ONLINE with a family member or close friend not living with you?” and “In the PAST MONTH how often have you had a VIRTUAL party or social gathering online with friends or family?” Answer options to all questions were (1) “never,” (2) “rarely,” (3) “sometimes,” (4) “often.”

7 The full list of denominations that was offered to respondents included Ultra-Orthodox or Haredi, Modern Orthodox, Conservative, Reconstructionist, Reform, Renewal, Humanist, Secular/Culturally Jewish, Just Jewish, no particular denomination, Other.

8 Using data available on the full population of 2020 Birthright applicants, respondents were weighted on gender, age, parental intermarriage, and whether or not the applicants had paid a deposit to participate in a Birthright trip. The weights, therefore, corrected for any nonresponse bias with respect to these variables. Although it is possible that differential nonresponse was correlated with other variables that could have biased results, experimental research has found low levels of nonresponse bias in surveys of Birthright applicants with response rates as low as 10% (Wright 2015).

9 Most (but not all) non-undergraduates in this sample were between the ages of 22 and 32.
